# Optimal Behavioral Hierarchy

**DOI:** 10.1371/journal.pcbi.1003779

**Published:** 2014-08-14

**Authors:** Alec Solway, Carlos Diuk, Natalia Córdova, Debbie Yee, Andrew G. Barto, Yael Niv, Matthew M. Botvinick

**Affiliations:** 1 Princeton Neuroscience Institute, Princeton University, Princeton, New Jersey, United States of America; 2 School of Computer Science, University of Massachusetts Amherst, Amherst, Massachusetts, United States of America; 3 Department of Psychology, Princeton University, Princeton, New Jersey, United States of America; Indiana University, United States of America

## Abstract

Human behavior has long been recognized to display hierarchical structure: actions fit together into subtasks, which cohere into extended goal-directed activities. Arranging actions hierarchically has well established benefits, allowing behaviors to be represented efficiently by the brain, and allowing solutions to new tasks to be discovered easily. However, these payoffs depend on the particular way in which actions are organized into a hierarchy, the specific way in which tasks are carved up into subtasks. We provide a mathematical account for what makes some hierarchies better than others, an account that allows an *optimal* hierarchy to be identified for any set of tasks. We then present results from four behavioral experiments, suggesting that human learners spontaneously discover optimal action hierarchies.

## Author Summary

In order to accomplish everyday tasks, we often divide them up into subtasks: to make spaghetti, we (1) get out a pot, (2) fill it with water, (3) bring the water to a boil, and so forth. But how do we learn to subdivide our goals in this way? Work from computer science suggests that the way a task is subdivided or decomposed can have a dramatic impact on how easy the task is to accomplish: certain decompositions speed learning and planning compared to others. Moreover, some decompositions allow behaviors to be represented more simply. Despite this general insight, little work has been done to formalize these ideas. We outline a mathematical framework to address this question, based on methods for comparing between statistical models. We then present four behavioral experiments, showing that human learners spontaneously discover optimal task decompositions.

## Introduction

Since the earliest days of psychology and neuroscience, a core objective within both fields has been to understand the formal structure of behavior [1]–[4]. In pursuing this question, both in humans and in other animals, a crucial and recurring observation has been that behavior displays a hierarchical organization. Simple actions fit together into coherent subtasks, which themselves combine to accomplish higher-level goals [5], [6]. This kind of tiered or nested structure is readily apparent in our everyday activities: Turning on the stove forms part of boiling water, which in turn forms part of cooking pasta. It has also been quantified in detailed formal analyses of behavior, both in the laboratory and in the field [7], [8].

The ubiquity of hierarchical structure in behavior presumably reflects an adaptive benefit. Consistent with this, computational analyses have revealed at least two important advantages that can be gained by organizing behavior hierarchically. First, hierarchical representations of behavior can be more compact or efficient than non-hierarchical (flat) representations, allowing complex behaviors to be encoded more economically at the neural level [9]. Second, hierarchical representations of action can facilitate the discovery of new adaptive behaviors, either through learning or through on-line planning [10]–[12] or problem-solving [13]–[16].

**Figure 1:**
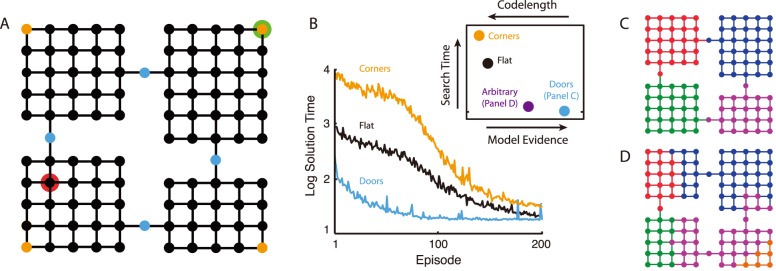
A. Rooms domain. Vertices represent states (green = start, red = goal), and edges feasible transitions. **B**. Mean performance of three hierarchical reinforcement learning agents in the rooms task. *Inset*: Results based on four graph decompositions. Blue: decomposition from panel C. Purple: decomposition from panel D. Black: entire graph treated as one region. Orange: decomposition with orange vertices in panel A segregated out as singleton regions. *Model evidence* is on a log scale (data range 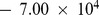 to 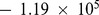). *Search time* denotes the expected number of trial-and-error attempts to discover the solution to a randomly drawn task or subtask (geometric mean; range 685 to 65947; tick mark indicates the origin). *Codelength* signifies the number of bits required to encode the entire data-set under a Shannon code (range 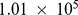 to 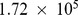). Note that the abscissa refers both to model evidence and codelength. Model evidence increases left to right, and codelength increases right to left. **C**. Optimal decomposition. **D**. An alternative decomposition.

An illustration of this latter point is provided in Figure 1. The example centers on an artificial reinforcement learning agent [17] that navigates from vertex to vertex in the grid shown in panel A. The agent must learn, through trial and error, to move from the start location highlighted in green to a rewarded goal location, highlighted in red. The black data-series in panel B charts the agent's improvement over successive trials. In contrast, the blue data-series tracks learning in a hierarchical reinforcement learning agent [11], [18]. This agent is furnished with subtask representations or subroutines for navigating to each of the “doorway” locations marked in blue in panel A (simulation code available online at www.princeton.edu/~matthewb). It can thus behave hierarchically, choosing among subroutines that in turn specify concrete, low-level actions. As is clear from the learning curve, the hierarchical agent converges on shortest-path behavior much more quickly than the flat agent.

Another way of interpreting this illustrative simulation is in terms of planning. In many models of planning (e.g. [19]), action plans are gradually refined based on a series of internal simulations, in each of which the outcomes of a potential line of behavior are projected. Interpreting the model in this way, the “trials” in Figure 1B correspond to successive internal simulations, and the effect of hierarchy is to reduce planning time.

While this example illustrates the point that hierarchy can facilitate the discovery of new adaptive behaviors, there is an important caveat: Not all hierarchies are created equal. The wrong hierarchical representation can actually undermine adaptive behavior. This point is again illustrated in Figure 1B. The orange data-series in the figure tracks the course of learning for a second hierarchical agent. This agent, like the one just considered, is furnished with a set of subroutines. However, here each subroutine involves navigating not to a doorway but into a corner (one of the locations highlighted in orange in panel A). In contrast to the doorway agent, this corner agent learns much more slowly than the flat agent. Obviously, it is not hierarchy *per se* that facilitates adaptive behavior. It matters very much which specific set of hierarchical representations an agent carries.

These observations bring to the surface a fundamental point concerning behavioral hierarchy: While hierarchy can facilitate learning, it also introduces a new learning problem, the problem of discovering beneficial rather than disruptive subtask representations.

Computational work in the area of hierarchical reinforcement learning has given rise to a number of approaches aimed at discovering useful behavioral hierarchies, leveraging ideas from information theory, graph theory, and developmental psychology [10], [11], [20]–[22]. For example, Simsek and Barto [20] describe a method based on betweenness, a graph centrality metric which measures the fraction of shortest paths that go through each vertex of a graph. They construct what they call an interaction graph, representing possible state transitions, and compute a weighted betweenness metric that depends on the costs associated with each path. Local maxima, which often appear in “bottleneck” states (described further below), represent subgoal locations that can be utilized in hierarchical representations. Van Dijk and Polani [21] take an information theoretic approach and define subgoals as states in which there is a significant change in the amount of relevant goal information, a measure of the amount of information that needs to be maintained about the goal at each step in order to perform well. Still other work has suggested that useful task decompositions might be learned through analyses of the causal structure of the environment, or via curiosity-driven learning mechanisms [22].

However, such work has never *directly* confronted the key underlying question of what exactly the agent should learn. Given that some hierarchies are better than others, can one specify for any given behavioral domain the best hierarchy overall? In other words, what would it mean for a behavioral hierarchy to be optimal?

It is this question that we confront in the present work. Our basic proposal is that the optimal hierarchy is one that best facilitates adaptive behavior in the face of new problems. We show how this notion can be made precise using the framework of Bayesian model selection. After presenting the formal framework, we present results from four behavioral experiments suggesting that human learners are able to discover decompositions deemed optimal in this way.

## Results/Discussion

### Formal approach

In order to set the stage, we briefly introduce some additional terminology from the reinforcement learning literature. The goal of a reinforcement learning agent is to find a reward maximizing *policy*, a mapping from states to actions, in an environment obeying certain Markovian dynamics. In particular, it is assumed the environment consists of a set of states, 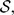 a set of actions, 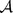, a transition function 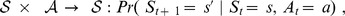 and a reward function 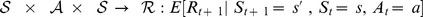, where 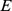 is expectation and 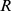 is scalar reward. There are several ways of incorporating hierarchy into reinforcement learning; we adopt the options framework approach [18] in this paper. An option may be thought of as a temporally extended action and consists of: an initiation set containing the states from which it may be invoked, a termination function 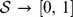 specifying the probability of terminating the option in each state, and a policy. Once invoked, the agent's behavior is controlled by the option-specific policy until it terminates, at which point the higher level policy again takes over. Options may also be nested, resulting in arbitrarily deep hierarchies. In this paper, we will use the terms option and subtask interchangeably. *Root-level* policy will refer to the policy at the top level (outside of all options), in contrast to option-level or subtask policies.

In any optimization problem, the crucial first step is to identify the objective. In the present case, this means asking: What exactly should an optimal hierarchy optimize? The rooms example in Figure 1 suggests a sensible answer to this question: An optimal hierarchy should maximize the efficiency with which an agent can discover new reward-maximizing behaviors. To make good on this idea, a method is needed for scoring or ranking candidate hierarchies on this property.

In order to solve this problem, we reframe it in terms of Bayesian model selection, where a set of candidate models are compared in their ability to account for a set of target data [23]. In the present case, the set of candidate models comprises all possible combinations of options with which the agent can be furnished. The data, in turn, are a target set of optimal behaviors (i.e. policies, a series of state–action pairs) representing the solutions to an *ensemble* of tasks faced by the candidate agent. That is, the agent is assumed to occupy a world in which it will be faced with a specific *set* of tasks in an unpredictable order, and the objective is to find a hierarchical representation that will beget the best performance on average across this set of tasks. An important aspect of this scenario is that the agent may *reuse* subtask policies across tasks (as well as task policies if tasks recur).

In what follows, we first describe how Bayesian model selection can be applied in this context. We then explain how model selection achieves the desired optimum, maximizing the ease with which adaptive behaviors can be discovered.

In Bayesian model selection, each candidate model is assumed to be associated with a set of parameters, and the fit between the model and the target data is quantified by the marginal likelihood or *model evidence*:
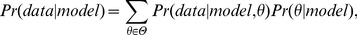
(1)where 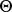 is the set of feasible model parameterizations. In the present setting, where the models in question are different possible hierarchies, and the data are a set of target behaviors, the model evidence becomes:
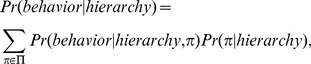
(2)where 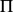 spans the set of behavioral policies available to the candidate agent, given its inventory of subtask representations (this includes the root policy for each task in the target ensemble, as well as the policy for each subtask itself). The optimal hierarchy is the one that maximizes the model evidence, as formulated in Equation 2.

Note that while the target behavior consists of the *optimal* (flat) policies specified as a series of state–action pairs, the parameter 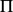 spans the range of *all* (hierarchical) policies the agent may be equipped with. Some of these will be compatible with the data, and some will not. Importantly, multiple settings of 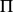 may be compatible with the data. In particular, the root-level policy for a state (in a particular task) is irrelevant if the state is covered by an option policy. The root policy is, in this setting, unconstrained, and can vary arbitrarily. This “freeing up” of parameters is critical in determining the optimal hierarchy.

In order to illustrate this approach, we consider an agent like the one in the rooms example from Figure 1: an agent whose actions equate to deterministic, reversible transitions between discrete states, visualizable as vertices in a graph. We assume, for concreteness, that the ensemble of tasks that the agent faces comprises the set of all shortest-path problems within the graph. In order to build an inventory of subtask representations, the agent is permitted to decompose the graph into a set of connected components (see Figure 1), defining regions within the state-space of its environment. The agent is then furnished with a subtask representation for each available method of transitioning between regions [24] (see Methods for further detail). For example, given the partitioning shown in Figure 1C, the rooms agent would obtain two subtask representations for each room, each with one doorway as its goal.

(Note that the foregoing exposition assumes that hierarchies are one level deep, and that the termination function for each option is non-zero in a single sub-goal state. This restriction was made for simplicity and for tractability in implementation. However, the general Bayesian model selection framework and optimality guarantees apply to arbitrary hierarchies without change.)

Applying Bayesian model selection under this problem formulation, the data to be modeled take the form of state–action pairs, where the states represent all of the shortest paths within the state-transition graph. In order to mark task boundaries, this concatenation is supplemented by a set of task-unique symbols, associated with indices specifying where each new task begins. The set of models (behavioral hierarchies) corresponds to the set of all possible decompositions of the graph. In this context, the model evidence assumes a surprisingly compact form:
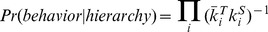
(3)where 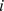 indexes vertex identifiers within the data; 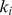 is the degree of the vertex appearing as data element i; 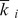 is 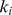 plus the number of subtasks initiable at 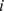; and 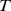 and 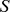 are indicator functions of 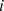, assuming a value (1 or 0) that indicates whether each element constrains the agent's task-level action policy (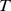) or a subtask-level policy (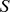). As detailed under Methods and in the online supplement, each of the terms in Equation 3 can be quantified based strictly on the target data and the graph itself.

**Figure 2:**
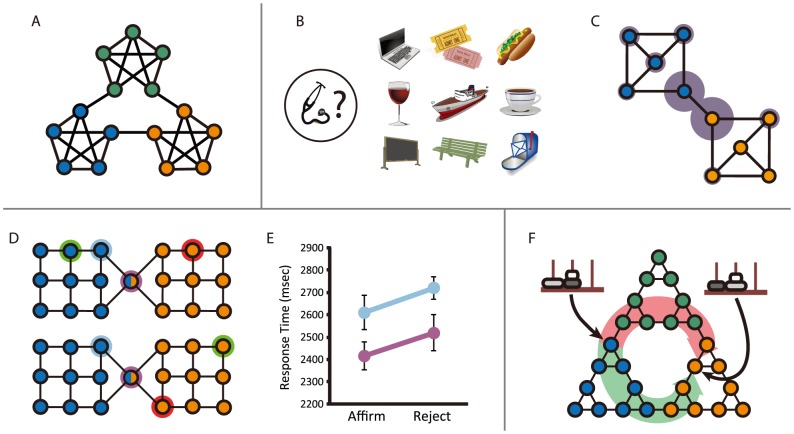
A. Graph studied by Schapiro et al. [41], showing the optimal decomposition. **B**. Task display from Experiment 1. Participants used the computer mouse to select three locations adjacent to the probe location. **C**. Graph employed in Experiment 1, showing the optimal decomposition. Width of each gray ring indicates mean proportion of cases in which the relevant location was chosen. **D**. Graph studied in Experiments 2 and 3, showing the optimal decomposition (two regions, with central vertex grouped either to left or right). *Top:* Illustration of a “delivery” assignment from Experiment 3 (green = start, red = goal), where bottleneck (purple) and non-bottleneck (blue) probes called for a positive response. *Bottom*: An assignment where bottleneck and non-bottleneck probes called for a negative response. **E**. Mean correct response times from Experiment 3. *Affirm*: trials where the probe fell on the shortest path between the specified start and goal locations. *Reject*: trials where it did not. Purple: bottleneck probes. Blue: non-bottleneck probes. **F**. State-transition graph for the Tower of Hanoi puzzle, showing the optimal decomposition and indicating the start and goal configurations of the kind studied in Experiment 4. A different set of colors was used for the beads in the actual experiment. Furthermore, as explained under Methods, the beads were the same size. The changes were made here for display purposes.

Figure 1B (inset) applies Equation 3 to the rooms domain, plotting the model evidence for four agent hierarchies. The hierarchy with the greatest evidence corresponds to the partition shown in Figure 1C. This partition, with subgoals corresponding to the doors, in fact represents the optimal behavioral hierarchy in this particular domain. Another example is shown in Figure 2F. This shows the task graph for the Tower of Hanoi, a puzzle in which disks must be moved from a start arrangement to a goal arrangement, without ever placing any disk upon a smaller one. The optimal hierarchy for this task divides the state space into three regions, each corresponding to one position of the largest disk.

Crucially, by maximizing the model evidence, these hierarchies also turn out to satisfy our original desideratum, maximizing the agent's ability to efficiently discover target behaviors. Specifically, the optimal hierarchy minimizes the geometric mean number of trial-and-error attempts necessary for the agent to discover the optimal policy for any selected task or subtask (see Figure 1B, inset, for illustrative data). An explicit proof of this point is provided in the online supplement. However, the conclusion follows from the fact that every candidate hierarchy induces a probability distribution over behaviors (see Eq. 2), and that the optimal hierarchy, by definition, places the greatest probability mass on the agent's target behavior. This further implies that the optimal hierarchy will minimize the number of trials needed, on average, to discover the target behavior.

It also happens that the optimal hierarchy, by maximizing the model evidence, is guaranteed to minimize the expected number of information-theoretic bits needed to specify a hierarchical policy consistent with the target data. That is, if we treat the target behavior as a stream of data, we can encode this stream using a set of symbols representing the top level and option policies (see e.g., [25], for a related example outside reinforcement learning). Depending on the set of options available, some encodings are more compact than others. The hierarchy that maximizes the model evidence induces an encoding that is the *most* compact. This once again follows directly from the fact that every candidate hierarchy induces a probability distribution over behaviors, and that the optimal hierarchy places the greatest probability mass on the target behavior. The optimal hierarchy will thus accord this behavior the shortest code length under a Shannon code assignment [26], also implying the shortest expected description for any task-specific behavior (i.e., shortest path).

Figure 1B (inset) shows the expected description length for several agent hierarchies in the rooms domain. As is clear from the figure, the hierarchy that maximizes the efficiency of representation also maximizes the efficiency of learning. This is no coincidence: It is a well established result from learning theory, echoed in empirical observations of human behavior, that ease of learning is directly related to descriptive complexity [27], [28]. Indeed, this connection has inspired previous efforts to identify useful subtask representations through data compression [21], [29]–[31].

A salient aspect of the specific hierarchies we have considered so far (Figures 1C, 2F), is that they carve the state-space at topological bottlenecks, narrow segments bridging between densely interconnected clusters of vertices. Further examples are shown in Figure 2, panels A, C, and D. The decompositions discovered here by Bayesian model selection strikingly resemble those arising from graph-theoretic algorithms for community detection, which explicitly aim to isolate tightly connected clusters within complex networks. Indeed, compression of walks on graphs has been employed as one method of community detection [25]. In the present case, where graph decompositions correspond to behavioral hierarchies, the prominence of bottlenecks is intuitive, in the sense that subtask representations are useful precisely to the extent that they carve tasks “at their joints.” Recognizing this parallel, some work in hierarchical reinforcement learning has used community structure in order to identify useful subtasks [20], [32], [33]. The present results place this past research on a normative basis, by showing that the behavioral hierarchies resulting from community or bottleneck detection approximate hierarchies that provably maximize the agent's ability to discover reward-maximizing behaviors. In fact, the two approaches are complementary: While the present work provides a normative basis for understanding which partitions are best, previous work on bottleneck detection offers heuristic algorithms that may find such partitions more efficiently than searching through the entire space of possible hierarchies. Of course, the approaches will not always coincide, and understanding how and when they differ is an interesting challenge for future work.

### Behavioral experiments

Having introduced a framework for identifying optimal behavioral hierarchies, we turn to the question of whether human learners decompose novel tasks in an optimal fashion. Some encouragement for this possibility comes from previous work in which related formal principles have been proposed to underlie learning in other domains, including vision [34], [35], working memory [36], [37], language [38], and others [39], [40]. Still more germane is a recent study in which participants were asked to parse sequences of visual stimuli whose ordering, unbeknownst to them, was determined by a random walk in the graph shown in Figure 2A [41]. Participants marked the transitions between the five-vertex clusters as natural breaking points, consistent with the idea that human sequence perception spontaneously detects temporal community structure.

In order to examine hierarchy learning in the context of goal-directed action, we conducted four new behavioral experiments. In each of these, undergraduate participants learned about and chose actions within graph-like domains. Our general prediction, probed in different ways in each experiment, was that participants would develop a hierarchical representation of each domain aligning with the one predicted by our theoretical framework. As in the rooms domain, the setup in all four experiments is that the agent is able to make deterministic reversible transitions between (discrete) states, and that the task ensemble consists of shortest path problems between all pairs of states. Although this is our present focus, it is not a general limitation of the framework. The optimality guarantees outlined above and detailed in the online supplement apply to arbitrary tasks.

In our first experiment, a group of forty participants prepared to make a set of “deliveries” by learning the layout of a small town. The town comprised a set of ten locations, each associated with a distinctive visual icon (Figure 2B). Participants were never given a bird's eye view of the town. Instead, during an initial training period, participants were drilled on the adjacency relations among individual locations. On each trial a randomly selected location was highlighted, and the participant's task was to select the three locations immediately adjacent to this probe (see Figure 2B). Following this training period, participants were informed that they would next be asked to navigate through the town in order to make a series of deliveries between randomly selected locations, receiving a payment for each delivery that rewarded use of the fewest possible steps. Before making any deliveries, however, participants were asked to choose the position for a “bus stop” within the town. Instructions indicated that, during the subsequent deliveries, participants would be able to “take a ride” to the bus stop's location from anywhere in the town, potentially saving steps and thereby increasing payments. Participants were asked to identify three locations as their first-, second- and third-choice bus-stop sites.

Crucially, the pattern of adjacencies to which participants were exposed was based on the graph shown in Figure 2C. As is obvious upon inspection, the graph has a single bottleneck at its center, and an optimal partition reflecting this fact (indicated by color in the figure). Bayesian model selection identifies two graph vertices, lying at this bottleneck, as optimal subgoal locations. Given the structure of the task and the goal of navigating rapidly to an *a priori* unknown location, the optimal strategy is to place the bus stop at one of these locations. The objective of the experiment was to evaluate whether participants could detect the bottleneck and exploit it in this way. It is important to stress that participants were never given a bird's-eye view of the town, or even direct information about relative Cartesian positions. The topology of the town graph had to be inferred solely from local adjacency information. Furthermore, all of the locations had exactly three neighbors and received on average equal exposure during training. There was thus nothing specially salient about any of them. Despite this challenge, participants showed a marked tendency to place the bus-stop at the locations predicted (see Figure 2C). After adjusting for chance, the two bottleneck locations were identified as first-choice locations 4.4 times as often as the remaining locations (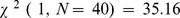, 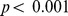). Among participants who were able at the end of the experiment to draw the underlying graph perfectly, 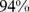 chose a bottleneck location first (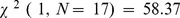, 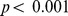).

The results of this initial experiment are consistent with the notion that human learners identify and exploit optimal task decompositions or behavioral hierarchies. However, it might be argued that the bus stop manipulation prompted a special, task-specific orientation. Two further experiments investigated whether human learners identify and exploit optimal hierarchies spontaneously, without such a prompt. In Experiment 2, ten participants completed a set of deliveries, with no mention of bus stops, within a town whose layout was based on the bottleneck graph in Figure 2D. Some deliveries were completed step by step, using a graphical interface that showed participants their current location and allowed them to select among adjacent locations. However, on another subset of trials participants were shown all town location icons concurrently and asked either to (1) indicate all locations lying on the shortest path between a specified start and goal *in any order*, or (2) identify any *single* location lying on this path. In the former condition, participants showed a strong tendency to select the bottleneck location first (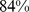 of correct responses on relevant trials; Monte Carlo test, 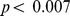). And in the single-location condition, participants again showed a strong tendency to select the bottleneck (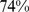 of correct responses on relevant trials; Monte Carlo test, 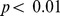). These findings suggest that participants planned their routes hierarchically, “thinking first” of transition-points between subregions, and then planning the specific steps needed to reach those transition points [42]. More importantly, the observed behavior confirms that participants decomposed the task space in an optimal fashion, consistent with the Bayesian model selection account.

These conclusions were reinforced by the results of a third experiment. Here, 21 participants made deliveries within a town based again on the graph from Experiment 2. Interleaved with step-by-step delivery trials like those in Experiments 1 and 2 were trials in which participants were presented with a start location and a goal location, and asked whether a third location would lie on the shortest path from one to the other (see Figure 2D). Correct response times were faster when the probe location lay at the boundary between subregions in the optimal parse than when it lay elsewhere in the graph (Figure 2D–E; 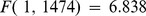, 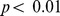), again consistent with the idea that route planning occurred initially at the level of the regions arising from the optimal decomposition, followed later by finer-grained selection. Further statistical analysis, detailed in the supplement, showed that this main effect was not explained by differences in probe frequency.

In a final experiment, we tested whether the predictions of the optimal hierarchy framework extend beyond the domain of spatial navigation. Here, we leveraged the Tower of Hanoi task. As shown earlier, the optimal decomposition of this task separates it into three regions (Figure 2F). Consider the problem defined by the start and goal states shown in Figure 2F. As also shown in the figure, there are two shortest-path solutions to this problem, each involving the same number of steps. The two paths differ, however, in terms of the number of boundaries they traverse between regions: One traverses one such boundary, the other two. Based on the idea that planning occurs first at the level of the regions defined by the optimal hierarchy, and that maintaining subgoals in memory is costly [43], we predicted that participants faced with this particular problem would prefer the path crossing only a single region boundary. This prediction was confirmed in an experiment involving thirty-five participants, who solved a series of Tower of Hanoi problems. When the problems of interest occurred, participants pursued the single-boundary solution in 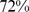 of cases (right-tail sign test, 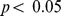). Seventeen subjects traversed the single-boundary route most often, while only seven showed the opposite asymmetry (one-tailed t-test, 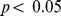).

The results of these four experiments support the conclusion that human learners discover optimal task decompositions and leverage these decompositions in planning action sequences. The data suggest that novel behavioral domains are spontaneously decomposed into subdomains or regions, and that planning initially focuses on transitions between these, typically via topological bottlenecks. More specifically, the decompositions selected are optimal in the sense specified in the Bayesian model selection account.

Although our focus has been on a reinforcement learning [17] characterization of learning and planning, this view includes more classic notions of planning both in artificial intelligence [14] and in psychology [16]. Such problems may be cast in the reinforcement learning framework (i.e. as Markov decision processes) by encoding the goal state in the reward function (e.g. by setting reward to be 0 in the goal state and -1 everywhere else). Although planning in reinforcement learning is often performed in the forward direction, when the goal state is isolable, one can also perform goal regression, serially satisfying a chain of preconditions. Subgoal discovery at the hierarchical level may help to determine the relevant preconditions, with the same optimality construct applying.

In psychology, a number of theorists have attempted to understand planning in the context of broader unified frameworks for cognition, such as ACT-R [13] and Soar [15]. In ACT-R, both goals and subgoals are specified by the task model. In Soar, subgoals specifically related to solving impasses in decision making are automatically acquired. The present paper outlines a normative framework for understanding task decompositions, and this information could in theory be applied to either ACT-R or Soar, specifying the type of decompositions each should *strive* to achieve.

This raises a final issue of note: It is not our proposal that human learners discover optimal hierarchies by literally computing the Bayesian model evidence given foreknowledge of target behaviors, as in Equation 3. The present experimental results thus raise the important question of what discovery procedure human learners actually employ in order to approximate the same result. One possible answer comes from recent work on statistical learning, which shows that simply learning to predict future events can support discovery of community structure and topological bottlenecks in novel behavioral domains [41]. An inviting direction for further work is to test whether this learning procedure might underlie the kind of hierarchy induction observed in the present experiments.

## Methods

### Computational analysis

#### Problem formulation

The Bayesian model selection approach compares agents equipped with different action hierarchies, but faced with the same ensemble of tasks. Our application of the approach focused on tasks taking the form of episodic Markov decision problems or MDPs [17]. A MDP comprises a set of states, a set of actions, a transition function that specifies the results proceeding from selection of specific actions in specific states, and a reward function attaching a scalar reward to each state transition. The challenge posed is to discover an action policy – a mapping from states to actions – that maximizes expected cumulative reward. For simplicity, we focused on MDPs with discrete, tabular representations of state, deterministic transition functions, and fully reversible actions (where reversibility means that for every action causing a transition from state 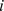 to 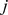, there is another action taking 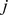 to 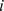). This focus allowed us to represent any particular problem domain in the form of an undirected graph, with a vertex for each state and edges marking feasible actions (i.e., transitions between states). Within this setting, we considered a task ensemble (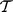) comprising the set of all shortest-path problems within the graph, each task being specified in terms of a start vertex (state), a goal vertex, and a negative reward associated with traversal of each edge (randomly sampled between 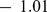 and 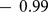, and held constant across tasks).

The agents considered under our model comparison approach were assumed to take the form of hierarchical reinforcement learning (HRL) agents implementing the options framework [18] (see also [11], [44]). Each agent was assumed to carry a set of policies, including (1) a deterministic root-level policy for each task in the target ensemble 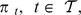 and (2) a fixed, agent-unique set of option policies 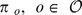, the same across all tasks. Note that, while the HRL framework includes learning procedures for tuning action policies at root and subtask levels, the “agents” we considered did not deploy such learning algorithms; they merely carried already optimal policies structured as in HRL.

As described earlier, the option set for each agent was fully determined by an agent-specific decomposition of the state-transition graph into connected components or regions. Following Hauskrecht et al. [24], we define a region's *entrance vertices/states* as the set of all vertices within the region that have at least one neighbor outside of it. Conversely, the *exit vertices/states* for a region comprise those vertices outside the region that receive at least one edge from a vertex inside it. Armed with these definitions, we viewed any region within a given graph decomposition as inducing a set of option representations, each having the region's entrance states plus the start state for the current task as its initiation set, the exit states for the region as its termination set, and one specific exit as its subgoal (see [11], [18] for a full explanation of these terms). Each option policy was assumed to cover only the states/vertices lying within the relevant region, with an action set including only atomic actions, i.e., transitions between neighboring states/vertices. The action set for root-level policies, in contrast, included options as well as atomic actions.

As introduced earlier, Bayesian model selection takes into account a dataset and a set of candidate models, each associated with a set of parameters (see Equation 1). In the present application, the target data comprise the set of behaviors representing solutions to the target task ensemble, here the set of all shortest paths within the state-transition graph. The set of models to be compared includes one HRL agent for each possible decomposition of the state-transition graph. Each candidate agent is associated with a policy set consisting of each task (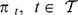) and option (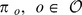) policy. The parameters, 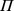, dictate the values of these policies, with a separate parameter specifying the optimal action for a single state in either the task or option policy. In the case of 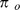, the number of possible values for each parameter equals the number of atomic actions available in the relevant state, which in turn is equal to the number of edges projecting from the corresponding vertex in the graph representation (i.e. the vertex degree). In the case of 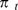, the number of possible values equals the number of atomic actions available plus the number of options available, a sum we designate as 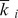. The number of options available corresponds to the number of exit states for the region: there is a separate option for navigating to each neighboring state in each adjacent region.

Note that the details of this application imply that option policies may only call primitive actions, and not other options. The depth of the behavioral hierarchy is thus limited to two levels. This restriction was adopted to assure computational tractability in the present application. However, it is important to note that the overall theoretical framework generalizes without any alteration to deeper hierarchies.

#### Calculation of model evidence

Given the above formulation, the model evidence can be written as in Equation 2. We assume a uniform distribution over model parameterizations. Thus, we can rewrite the model evidence as follows:
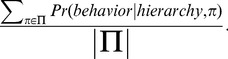
(4)


We assume the parameter space to span only deterministic policies. This means that any specific model parameterization will be either perfectly compatible with the data (i.e., 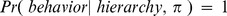) or categorically incompatible (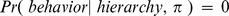). If we denote the set of all compatible parameterizations as 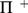, then
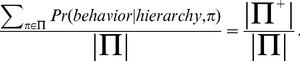
(5)


Thus, in order to calculate the model evidence, it suffices to determine the proportion of all parameterizations that are compatible with the data.

Imagine starting with all of the parameters unset. The number of possible parameterizations, 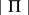, is equal to the product of the number of values that each parameter can take. The target data then arrive sequentially, one vertex–action pair at a time. Because the policy parameters for the relevant task must specify the actions that actually occur in the data, each newly arriving element has the potential to reduce the set of compatible parameterizations. That is, each element of the data has the potential to fix the value of one or more parameters, ruling out the remaining inconsistent values. Mathematically speaking, the consequence is to divide the number of compatible parameterizations by the number of values the parameter can take. If we designate this number as 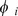, with 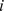 indexing the data element, then the number of compatible parameterizations is
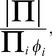
(6)and the model evidence is thus
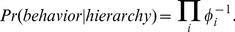
(7)


All that remains is to specify how 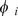 should be chosen for any element 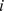. To see this, consider that any state transition in the data can constrain (1) the root policy 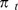 for the current task, (2) an option-specific policy 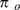, (3) both the root policy and an option policy, or (4) neither. Because each state-specific parameter in 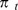 can take on 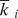 values, case (1) requires 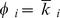. Because each state-specific parameter in 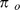 can take on 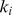 values, case (2) requires 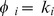. It naturally follows that case (3) requires 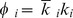. In case (4), where no constraint is added, 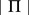 should remain unchanged, thus requiring 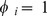. Taking these points on board,
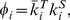
(8)where 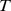 and 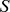 are indicator functions of 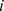: 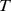 assumes value 1 if data-element 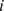 imposes a constraint at the root (task) level; 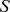 does so if the element imposes a constraint at the option (subtask) level; and each is otherwise zero. One further requirement we impose is that if a subsequence can be represented by an option, then the option policy is invoked (constrained) rather than the root-level policy (except on the first step, where both policies are constrained). This assumption is mild, but necessary for the optimality arguments to logically follow: If an agent is equipped with an option, it is assumed the option is used and not ignored.

The online supplement illustrates calculation of the model evidence through a concrete example. Also presented in the supplement are formal proofs of the optimality assertions advanced in the main body of the paper.

#### Ancillary procedures

As noted earlier, shortest paths were generated by adding a small amount of frozen noise to the edge weights. This approach was taken to avoid ties and to assure that the same path would always be followed between any two nodes, a condition necessary in order for deterministic option policies to transfer between tasks. We found that for some but not all graphs tested, the optimal partition varied slightly depending on the choice of shortest paths. For example, in the rooms domain (Figure 1), although the optimal hierarchy always separated out the four rooms at the doorways, it sometimes also included an isolated singleton near the center of one of the rooms. This coincides with previous work grounded in information theory, which suggests that in addition to bottlenecks, such locations are salient because they correspond to local maxima in goal information transitions [21], [45]. However, such results were idiosyncratic and appeared to arise from shortest-path choices that happened to channel behavior across small sets of edges, effectively creating behavioral bottlenecks not reflected in the topology of the graph itself. Because this is not a general property, it is unlikely to be reflected in the mean effects across participants that we focus on in the present paper. We thus do not pursue this point further. Instead, in order to avoid this nuisance effect, we searched for sets of shortest paths whose edge-traversal statistics closely approximated the betweenness-centrality statistics of the set of all edges in the underlying graph, quantifying the goodness of fit using Euclidean distance. This optimization was performed based on a sample of 100,000 shortest path sets. That is, for each graph, we first computed the edge betweenness statistics based on all possible shortest paths, and then separately based on each candidate set of consistent shortest paths (as described above). The set of edge betweenness statistics based on each set of paths was treated as a point in a high dimensional space, and the set of consistent shortest paths closest to the set of all paths in this space was selected.

In order to search the space of partitions for the partition yielding the highest model evidence, we followed Brandes et al. [46] and reformulated the problem as binary integer programming. In this formulation, the optimization is over a set of binary variables, with one variable for each edge in the graph. Setting a variable to ‘1’ turns the corresponding edge ‘on’, and the connected components correspond to separate communities. We used a genetic algorithm to search the resulting space, as implemented by the GA library in R [47]. Each generation consisted of 2000 individuals. The search was halted when the best score remained unchanged for 20 generations. Because genetic algorithms are not guaranteed to find the global optimum, we ran the search for each graph multiple times, each time with a different starting population. The space of partitions for each of the graphs from the navigation experiments and for the graph from Schapiro et al. [41] (see Figure 2A) was searched 1000 times. The space of partitions for the Tower of Hanoi graph was searched 500 times, and that for the rooms graph was searched 20 times.

In Experiments 2-3 the optimal parse involved two regions, with the bottleneck vertex assimilated to one. However note that, given the graph's symmetry, this implies the existence of two parses with equal model evidence: One incorporating the bottleneck vertex into one region, the other parse incorporating it into the other region. Figure 2D was designed to reflect this tie outcome.

### Behavioral experiment 1

Ethics statement: All experimental procedures, including procedures for informed consent, were approved by the Princeton University Institutional Review Board.

#### Subjects

Forty adults from the Princeton community (21 female; ages 18–21) participated. All gave written consent and were either given course credit or a nominal payment for their participation.

#### Materials and procedure

Participants were told that they were going to navigate through a virtual town to make deliveries. Each in a set of locations (icons) was mapped to a node in a graph (see Figure 2C). The present experiment began with an extended training phase, which took place before any deliveries were actually assigned. During each trial within the training period, participants saw all ten location icons in a randomly sorted array (see Figure 2B). One of the locations was highlighted, and the participant was asked to identify that location's three immediate neighbors in the town, using mouse clicks, in a self-paced fashion. Three selections were permitted. Each icon selected was immediately circled in green if it was a neighbor, and in red if not. If three correct selections were made, a new location was highlighted (sampled randomly without replacement), initiating the next trial. If, after each three selections, any selection was incorrect, the original index item remained highlighted while the rest of the display was reinitialized, and the participant made another attempt at identifying the relevant neighbors. When participants managed to identify all three neighbors correctly on the first round of any trial, they received a “point.” The training phase continued for a total of 55 minutes (for the first twenty participants) or 40 minutes (for remaining participants). The time was reduced partway through the experiment because we noticed that performance was at ceiling for most participants after 40 minutes.

Following the training phase, the experimenter introduced the delivery task, informing participants that deliveries would involve randomly selected initial and target locations, and that a “point” would be awarded for deliveries completed in the fewest possible steps. Participants were shown an example display, which showed icons indicating the current location, goal location and all locations adjacent to the current location, and 20 subjects completed a *single* practice delivery trial, using key presses to transition from the current location to an adjacent location until the goal was reached. (This practice trial was omitted for the remaining subjects based on a late-coming concern that the sequence of actions it elicited might bias later behavior. However, no difference was ultimately observed between the behavior of participants who completed the practice trial and participants who did not.)

The notion of a bus-stop was also introduced at this point. Participants were told that before embarking on the delivery task, they would be asked to position a bus stop within the town. They were told that a well-chosen location could help them navigate efficiently. During subsequent delivery trials, the participant was informed, their chosen location would appear in the display, and they could transition to it in one step, at any time, by pressing the 0 key. If they used the bus-stop to complete the delivery in fewer steps than the shortest path attainable without a bus-stop jump, they would receive an additional bonus point for the delivery.

Following provision of this information, participants were asked to provide their first, second and third choices for the bus-stop location. Participants then completed three delivery trials, to confirm that they had understood the task description. At the close of the experiment, participants were asked to draw a map of the town in the form of a graph, with nodes representing locations and edges indicating adjacency relations.

Supplementary results from this and the subsequent experiments are reported in the online supplement.

### Behavioral experiment 2

#### Subjects

Ten adults from the Princeton community (5 female, ages 18–21) participated. All gave written consent and were either given course credit or a nominal payment for their participation.

#### Materials and procedure

As in Experiment 1, participants were told that they were going to navigate through a virtual town to make deliveries. And once again, each in a set of locations (icons) was mapped to a node in a graph, in this case a graph of size nineteen (see Figure 2D). The experiment consisted of 19 blocks. It began with 6 blocks of 6 deliveries each. Each delivery trial was exactly as in Experiment 1, but without the bus-stop destination. In order to ensure proper learning of the town structure, this time participants were aided by periodic displays of a birds-eye view of the town's underlying map. Note that this visual aid makes bottleneck discovery straightforward. However, in this experiment we were not interested in bottleneck discovery per se, but rather in the question of whether participants used this knowledge to plan action sequences hierarchically. In the first 6 blocks, participants could look at the map as long as they wished. From the end of block 7 through the end of the task, they could look at the map for 3 seconds. This latter design decision was imposed with the assumption that participants should already be familiar with the town layout in later trials, reducing the time spent looking at the map and allowing for more trials to be collected.

Starting at the end of block 6 and through block 19 (the last block), some of the trials were normal delivery trials, and some were “path identification” tasks (the type of trial was determined with probability 0.5). In “path identification” trials participants were shown a grid with all 19 locations in random order with a start location identified with a green box and a target location with a red box. In 40% of these trials, participants were asked to identify, using mouse-clicks, all the locations that would lie on a shortest path between the start and target, in any order. Participants could choose locations by clicking on them and un-choose them by clicking again. Chosen locations were marked by a gray square around them. The trial ended either when the chosen locations formed a shortest path, or after a maximum of 15 clicks. In the other 60% of “path identification” trials, participants were shown the grid with the start and target locations and asked to click on just one location that lay in some shortest path between them. The trial ended after one click, regardless of whether the choice was correct or not. In all path identification trials participants received feedback indicating whether their choices were correct. A 40/60 split was chosen because the trials on which participants were asked for the full path were significantly longer in duration, limiting the number of data points that we would be able to collect overall.

### Behavioral experiment 3

#### Subjects

Twenty-one adults from the Princeton community (11 female, ages 18–21) participated. All gave written consent and were either given course credit or a nominal payment for their participation.

#### Materials and procedure

As in Experiments 1 and 2, participants were told that they were going to navigate through a virtual town to make deliveries. And once again, each in a set of locations (icons) was mapped to a node in a graph, in this case the graph of size nineteen used in Experiment 2 (see Figure 2D). Following the structure of Experiment 2, this experiment began with 6 blocks of 6 deliveries each, followed by a birds-eye view of the town's map, to aid in learning the town's distribution. At the end of these first 6 blocks, participants could look at the map as long as they wished. From the end of block 7 through the end of the task, they could look at the map for 3 seconds.

Each delivery trial was exactly as in Experiment 1 and 2. From block 6 through block 19 (the last block), at the end of each set of deliveries participants were asked ten Yes/No questions of the form “If you had to navigate from A to B, would you go through C?” Locations A, B and C were depicted graphically using their corresponding icon. The questions of interest were chosen randomly from a pool of four types, plus some extra filler questions: queries could be about local (A and B on the same side of town) or non-local (A and B on opposite sides) locations, and they could be about the bottleneck (C corresponding to bottleneck location) or about another non-bottleneck node. Type 1 queries were about non-local deliveries and the probe node C was the bottleneck (therefore the correct answer was always Yes). Type 2 queries were non-local deliveries and the through node was either of the nodes adjacent to the bottleneck on the target side of town (correct response was always Yes). Type 3 and Type 4 queries were local ones (A and B on same side), with or without the bottleneck as the through node, respectively (correct response was always No). A set of extra filler queries involved local deliveries, sometimes with the bottleneck as either start or target, with the through node selected from the same side of the city (correct answer could be Yes or No, depending on the participant's choice of shortest path).

All response times faster than 250 msec or slower than 7000 msec were excluded, and the remaining response times were log-transformed. Participants answered the queries correctly 98% of the time, and we excluded from our analyses the few incorrect responses. Our central predictions were that in queries where the bottleneck was the queried through node (Types 1 and 3), participants would be faster to correctly respond Yes or No than in queries involving the adjacent nodes (Types 2 and 4).

### Behavioral experiment 4

#### Subjects

Thirty-five adults from the Princeton University community (15 female, ages 18–46) participated in this study. All gave written consent and received a nominal payment for their participation.

#### Materials and procedure

Participants were trained to perform a computer-based version of the three-disk Tower of Hanoi (ToH) puzzle. The display showed a rectangular base supporting three posts, with three beads (isoluminant in red, green and blue) threaded onto the posts. Participants solved a series of puzzles, moving beads from post to post, one at a time, to transform initial configurations into target configurations. To move a bead, keys corresponding to its current and desired new positions were pressed in series (the J, K, and L keys and right index, middle and ring fingers were used for this purpose). In addition to the current bead configuration, the display also included an image of the goal configuration in the upper right portion of the screen.

Participants were required to follow a set of rules restricting the range of legal moves. Specifically, if the three colors are designated C1, C2 and C3, the rules specified that C2 could never be placed on top of C1 and that C3 could never be placed on top of either C1 or C2. The specific colors assigned to these three roles was counterbalanced across subjects. (The standard ToH task involves disks of different diameters rather than different colors; we used colors in preparation for a follow-up fMRI study, where considerations of visual similarity will be important). If an illegal move was attempted, a brief tone was sounded and no change would occur in the display.

After an initial orientation, participants performed a series of randomly selected ToH problems consisting of random start and goal configurations. This phase of the session lasted twenty minutes and was entirely self-paced. No limit was imposed on the number of moves allowed. However, participants received a monetary bonus of 2 cents for each puzzle solved, and were rewarded with a performance bonus of 3 cents for reaching the goal state in the minimum numbers of moves. At the end of each game, subjects were informed of their earnings (e.g., “You have earned 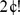
”). Participants then pressed the space bar to begin a new trial.

## Supporting Information

Text S1 Includes a step-by-step example of how to calculate the model evidence, the optimality proofs described in the main text, and supplementary experimental results.
(PDF)

## References

[1] Hebb DO (1949) The organization of behavior: A neuropsychological theory. New York, NY: John Wiley & Sons.

[2] Miller GA, Galanter E, Pribram KH (1960) Plans and the structure of behavior. New York, NY: Holt, Rinehart & Winston.

[3] Tinbergen N (1951) The study of instinct. Oxford, England: Clarendon Press.

[4] Tolman EC (1932) Purposive behavior in animals and men. New York, NY: Century.

[5] Botvinick MM (2008) Hierarchical models of behavior and prefrontal function. Trends in Cognitive Sciences 12: 201–208. 18420448 10.1016/j.tics.2008.02.009 PMC2957875

[6] Shallice T, Cooper RP (2011) The organization of mind. Oxford, England: Oxford University Press.

[7] Whiten A, Flynn E, Brown K, Lee T (2006) Imitation of hierarchical action structure by young children. Developmental Science 9: 574–582. 17059454 10.1111/j.1467-7687.2006.00535.x

[8] Zacks JM, Kurby CA, Eisenberg ML, Haroutunian N (2011) Prediction error associated with the perceptual segmentation of naturalistic events. Journal of Cognitive Neuroscience 23: 4057–4066. 21671745 10.1162/jocn_a_00078 PMC8653780

[9] Graybiel AM (1998) The basal ganglia and chunking of action repertoires. Neurobiology of Learning and Memory 70: 119–136. 9753592 10.1006/nlme.1998.3843

[10] Barto AG, Konidaris GD, Vigorito CM (2013) Behavioral Hierarchy: Exploration and Representation, Springer. pp. 13–46.

[11] Botvinick MM, Niv Y, Barto AC (2009) Hierarchically organized behavior and its neural foundations: A reinforcement learning perspective. Cognition 113: 262–280. 18926527 10.1016/j.cognition.2008.08.011 PMC2783353

[12] Foster D, Dayan P (2002) Structure in the space of value functions. Machine Learning 49: 325–346.

[13] Anderson JR, Bothell D, Byrne MD, Douglass S, Lebiere C, et al (2004) An integrated theory of the mind. Psychological Review 111: 1036–1060. 15482072 10.1037/0033-295X.111.4.1036

[14] Boutilier C, Dean T, Hanks S (1999) Decision-theoretic planning: structural assumptions and computational leverage. Journal of Artificial Intelligence Research 11: 1–94.

[15] Laird JE (2012) The Soar cognitive architecture. Cambridge, MA: The MIT Press.

[16] Newell A, Simon HA (1972) Human problem solving. Englewood Cliffs, NJ: Prentice-Hall.

[17] Sutton RS, Barto AG (1998) Reinforcement learning: An introduction. The MIT Press.

[18] Sutton RS, Precup D, Singh S (1999) Between MDPs and semi-MDPs: A framework for temporal abstraction in reinforcement learning. Artificial Intelligence 112: 181–211.

[19] Sutton RS (1990) Integrated architectures for learning, planning, and reacting based on approximating dynamic programming. In: Proceedings of the 7th International Conference on Machine Learning. pp. 216–224.

[20] Simsek Ö, Barto AG (2008) Skill characterization based on betweenness. In: Koller D, Schuurmans D, Bengio Y, Bottou L, editors, Advances in Neural Information Processing Systems. volume 21 , pp. 1497–1504.

[21] van Dijk SG, Polani D (2011) Grounding subgoals in information transitions. In: IEEE Symposium on Adaptive Dynamic Programming And Reinforcement Learning (ADPRL). IEEE, pp. 105–111.

[22] Vigorito CM, Barto AG (2010) Intrinsically motivated hierarchical skill learning in structured environments. IEEE Transactions on Autonomous Mental Development 2: 132–143.

[23] MacKay DJC (2003) Information theory, inference and learning algorithms. Cambridge University Press.

[24] Hauskrecht M, Meuleau N, Kaelbling LP, Dean T, Boutilier C (1998) Hierarchical solution of Markov decision processes using macro-actions. In: Proceedings of the Fourteenth Conference on Uncertainty in Artificial Intelligence. San Francisco, CA: Morgan Kaufmann, pp. 220–229.

[25] Rosvall M, Bergstrom CT (2008) Maps of random walks on complex networks reveal community structure. Proceedings of the National Academy of Sciences 105: 1118–1123. 10.1073/pnas.0706851105 PMC2234100 18216267

[26] Cover TM, Thomas JA (2012) Elements of information theory. Hoboken, NJ: John Wiley & Sons.

[27] Feldman J (2000) Minimization of boolean complexity in human concept learning. Nature 407: 630–633. 11034211 10.1038/35036586

[28] Kemp C (2012) Exploring the conceptual universe. Psychological Review 119: 685–722. 22924770 10.1037/a0029347

[29] Schmidhuber J (1992) Learning complex, extended sequences using the principle of history compression. Neural Computation 4: 234–242.

[30] Thrun S, Schwartz A (1995) Finding structure in reinforcement learning. In: Tesauro G, Touretzky DS, Leen TK, editors, Advances in Neural Information Processing Systems, volume 7 . pp. 385–392.

[31] van Dijk SG, Polani D, Nehaniv CL (2011) Hierarchical behaviours: getting the most bang for your bit. In: Kampis G, Karsai I, Szathmáry E, editors, Advances in Artificial Life: Darwin Meets von Neumann, Springer. pp. 342–349.

[32] Kazemitabar SJ, Beigy H (2009) Automatic discovery of subgoals in reinforcement learning using strongly connected components. In: Köppen M, Kasabov N, Coghill G, editors, Advances in Neuro-Information Processing. Springer, volume 5506 , pp.829–834.

[33] Moradi P, Shiri ME, Rad AA, Khadivi A, Hasler M (2012) Automatic skill acquisition in reinforcement learning using graph centrality measures. Intelligent Data Analysis 16: 113–135.

[34] Feldman J (2009) Bayes and the simplicity principle in perception. Psychological Review 116: 875–887. 19839688 10.1037/a0017144

[35] Orbán G, Fiser J, Aslin RN, Lengyel M (2008) Bayesian learning of visual chunks by human observers. Proceedings of the National Academy of Sciences 105: 2745–2750. 10.1073/pnas.0708424105 PMC2268207 18268353

[36] Brady TF, Konkle T, Alvarez GA (2009) Compression in visual working memory: Using statistical regularities to form more efficient memory representations. Journal of Experimental Psychology: General 138: 487–502. 19883132 10.1037/a0016797

[37] Mathy F, Feldman J (2012) What's magic about magic numbers? Chunking and data compression in short-term memory. Cognition 122: 346–362. 22176752 10.1016/j.cognition.2011.11.003

[38] Finley S, Newport EL (2010) Morpheme segmentation from distributional information. In: Boston University Conference on Language Development (BUCLD) Online Proceedings Supplement.

[39] Chater N, Vitányi P (2003) Simplicity: A unifying principle in cognitive science? Trends in Cognitive Sciences 7: 19–22. 12517354 10.1016/s1364-6613(02)00005-0

[40] Robinet V, Lemaire B, Gordon MB (2011) MDLChunker: A MDL-based cognitive model of inductive learning. Cognitive Science 35: 1352–1389. 21824175 10.1111/j.1551-6709.2011.01188.x

[41] Schapiro AC, Rogers TT, Cordova NI, Turk-Browne NB, Botvinick MM (2013) Neural representations of events arise from temporal community structure. Nature Neuroscience 16: 486–492. 23416451 10.1038/nn.3331 PMC3749823

[42] Wiener JM, Mallot HA (2003) ‘Fine-to-coarse’ route planning and navigation in regionalized environments. Spatial Cognition & Computation 3: 331–358.

[43] Anderson JR, Douglass S (2001) Tower of Hanoi: Evidence for the cost of goal retrieval. Journal of Experimental Psychology: Learning, Memory, and Cognition 27: 1331–1346. 11713870

[44] Barto AG, Mahadevan S (2003) Recent advances in hierarchical reinforcement learning. Discrete Event Dynamic Systems 13: 341–379.

[45] van Dijk SG, Polani D (2013) Informational constraints-driven organization in goal-directed behavior. Advances in Complex Systems 16: 1350016.

[46] Brandes U, Delling D, Gaertler M, Gorke R, Hoefer M, et al (2008) On modularity clustering. IEEE Transactions on Knowledge and Data Engineering 20: 172–188.

[47] Scrucca L (2013) GA: a package for genetic algorithms in R. Journal of Statistical Software. 53: 1–37.

