# Supplementary material for: Optimal Behavioral Hierarchy
Source: PLoS Comput Biol. 2014 Aug 14;10(8):e1003779. doi: 10.1371/journal.pcbi.1003779 (PMC4133163; doi:10.1371/journal.pcbi.1003779)
Supplement: Text S1 — Includes a step-by-step example of how to calculate the model evidence, the optimality proofs described in the main text, and supplementary experimental results. (PDF) [file pcbi.1003779.s001.pdf]

Supplementary methods and results

Computational analysis

Calculation of model evidence

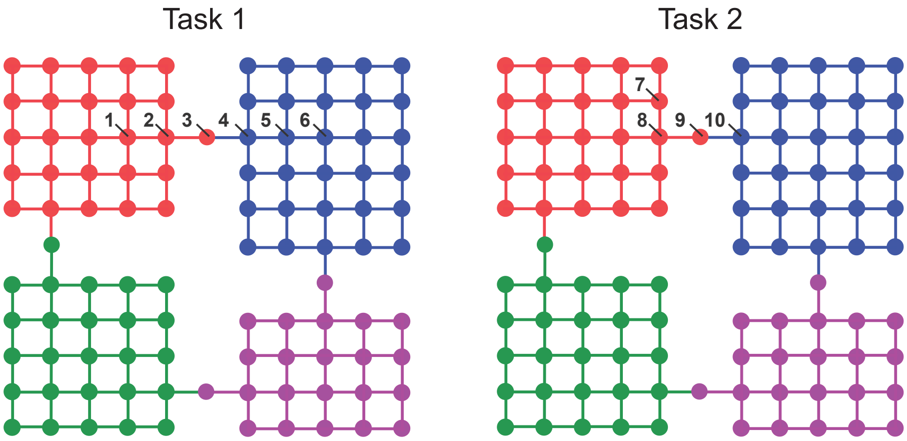

Figure S1. Calculating model evidence example

The procedure employed to calculate the model evidence is described in detail in the main text. Here we provide a concrete illustration. To this end, we revisit the rooms domain and consider scoring the particular partition shown in Figure S1. Imagine that the data begin with the two task-specific paths shown in that figure. Recall that in general, the data consist of optimal *policies*. When transitions are deterministic, the data may equivalently consist of a sequence of state transitions; the optimal policies are then implied. In what follows, we take the latter view to simplify the exposition.

Consider Task 1 first. The node appearing first on the path has four primitive actions available (north, south, east, west) and two options (go to blue region, go to green region). We can begin coding this path by considering taking the action *east* from element 1. However, as noted in the main text, our coding scheme assumes that if an option is available, it will be used. Because the option *go to blue region* also applies, it is selected. The transition from element 1 to 2 thus constrains both the task-specific policy π_{t1} (setting it to the option *go to blue region*) and the option-specific policy π_o for the *go to blue region* option (setting it to the action *east*). We compute ϕ_1 using Equation 8 from the main text. Because there are six choices at the top level (four actions and two options), $\overline{k}_1$ is 6, and because there are four choices at the option level (just the four actions), k_1 is 4. We thus have $\phi_1 = \overline{k}_1 k_1 = 6 \cdot 4 = 24$.

The transitions from element 2 to 3 and from 3 to 4 are guided by the option selected at element 1 (recall that behavior continues to be controlled by an option until it terminates, which in this case happens only when the subgoal in the blue region is reached). Consequently, these steps further constrain the option policy, but not the root level policy ($\phi_2 = k_2 = 4$, $\phi_3 = k_3 = 2$). The root level policy must again be invoked in the transition from element 4 to 5, setting $\overline{k}_4$ to 6 (the standard four actions are available, as are the options *go to red region* and *go to purple region*). However, because the ensuing sequence does not exit the blue region, no option can be selected, and the policy is instead set to the primitive action *east*. The transition from 5 to 6 is also guided by the root level policy, and $\phi_5 = \overline{k}_5 = 4$ (recall that the initiation set for options includes only the entrance states; there are thus no options available).

Element 7 begins a new task, and once again it is evident that an option is selected. Indeed, it is the same option that was selected at the outset of Task 1 (the sequence leads to the same exit node 10). The transition from 7 to 8 thus constrains both the root (setting it to the option *go to blue region*) and option (setting it to the action *south*) policies, and $\phi_7 = \overline{k}_7 k_7 = 5 \cdot 3 = 15$. On the transitions from 8 to 9 and 9 to 10, the same option remains in control. It is important to note, however, that these steps do *not* constrain this option's policy. The reason is that these same steps were taken, under the same option, in Task 1 (transitions 2-3 and 3-4). Because the option, like all options, can be reused across tasks, the constraints imposed on the option policy by those earlier steps already assure consistency with the repeated transitions in Task 2. Since these steps do not impact Π^+, $\phi_8 = \phi_9 = 1$.

Calculation of the factors ϕ_i would continue from this point, spanning all subsequent tasks contained in the dataset. The model evidence would then be directly computable, using Eq. 7.

Optimality

We show here that by choosing an action hierarchy that maximizes the model evidence, one maximizes the agent's ability to discover adaptive behaviors. We assume that the agent must discover the solution to each task only once (the first time that task is confronted), after which the solution can be stored and reused. Task frequency is thus not a determinative factor.

The adaptive behaviors in question span two levels. First there is the challenge of discovering an optimal root-level policy for each task in the relevant target ensemble. Second, because the resulting set of root level policies will in general call options, there is the additional challenge of learning optimal policies for those options themselves. The problem of discovering adaptive behaviors can thus be decomposed

into a set of sub-problems, one for each task (the challenge at the task level is to discover the optimal root-level policy, *given* options already furnished with optimal policies) plus one for each option. We refer to the union of these sub-problems as the *target set*. We aim to show that the hierarchy that maximizes the model evidence maximizes the expected log probability that the agent, proceeding by trial and error, will discover the optimal policy for a problem sampled randomly from the target set, prior to the arrival of any chosen deadline.

We begin with the model evidence itself: $Pr(behavior|hierarchy)$. Note that this can be regarded as the probability that the agent will generate the target behavior (the behavior described in the target data) based on simple trial-and-error. Each action in the process is generated by randomly selecting one value for the relevant policy parameter. Taking on board the hierarchical structure of the available policies, the model evidence can be thought of as:

$$Pr(behavior|hierarchy) = \prod_{t \in \mathcal{T}} p_t \prod_{o \in \mathcal{O}} p_o, \tag{1}$$

where p_t is the probability of randomly guessing the correct root-level policy for task t, and p_o is the probability of randomly guessing the correct option-level policy for option o. Taking the log on both sides gives,

$$\log Pr(behavior|hierarchy) = \sum_{t \in \mathcal{T}} \log p_t + \sum_{o \in \mathcal{O}} \log p_o = \sum_{j \in \mathcal{T} \cup \mathcal{O}} \log p_j. \tag{2}$$

Dividing by the size of the target set,

$$\frac{\log Pr(behavior|hierarchy)}{|\mathcal{T} \cup \mathcal{O}|} = \frac{\sum_{j \in \mathcal{T} \cup \mathcal{O}} \log p_j}{|\mathcal{T} \cup \mathcal{O}|}, \tag{3}$$

which, because we assume random sampling from the target set, implies

$$\log Pr(behavior|hierarchy) \propto E[\log p_{j \in \mathcal{T} \cup \mathcal{O}}]. \tag{4}$$

Thus, maximizing the model evidence also maximizes the expected log probability of correctly guessing the contents of a root or option level policy drawn at random from the target set. This will obviously remain true if multiple independent guesses are permitted. It is also easy to show, by extension, that maximizing

the model evidence minimizes the geometric mean number of trial-and-error attempts necessary for the agent to discover the optimal policy for a task or option randomly drawn from the target set. Relating the present theoretical approach to more sophisticated procedures, such as temporal-difference learning or Monte Carlo tree search, is an objective for ongoing work.

As asserted in the main text, maximizing the model evidence is also optimal in a second sense: It minimizes description length. Specifically, it minimizes the number of information-theoretic bits necessary to describe optimal behavior, given an ensemble of target tasks. “Description” here means specifying the policy for each task and each option, indicating which particular action should be taken in each state. From the definition of ϕ_i in the preceding section, it follows that the number of bits required by element i of the data is $\log_2 \phi_i$. The number of bits needed to describe the entirety of the data is therefore

$$-\log_2 \left(\prod_i \phi_i^{-1} \right), \tag{5}$$

where i ranges over data elements. The expression in parentheses here is the model evidence (see Eq. 7). Thus, maximizing the model evidence minimizes the number of bits necessary to specify a policy for the agent that is consistent with the data.

Although our focus has been on problems with deterministic reversible transitions, a similar set of arguments apply to the stochastic case. As described previously, the data consist of optimal task policies. With deterministic transitions, the policies have to be defined only for states along the shortest paths, as the agent cannot be knocked off-course. In the general case, the optimal policy has to be defined for all states that have a greater than zero percent probability of being visited. The arguments above then naturally follow. Maximizing the model evidence aids the discovery of optimal policies on average across tasks, and minimizes the number of bits necessary to store these policies.

Supplementary experimental results

Experiment 1

During the training phase, participants cycled through the full set of locations an average of 18.3 times. Of the forty participants, 23 (58%) identified one of the two bottleneck locations as their first bus-stop choice, far above the number that would be predicted to occur by chance, $\chi^2(1, N = 40) = 35.16$, $p < 0.001$. Additional analyses indicated that bus-stop choices did not differ significantly with training

duration (40 vs. 55 minutes). Of the 15 participants who selected the two bottleneck locations as their first two bus-stop choices, 11 selected an adjacent node – that is, one of the nodes with the highest graph centrality among those available – as their third choice. Two of the remaining participants violated the task instructions in order to once again select a bottleneck location. Participants who made no errors on the final two cycles were classified as highly successful learners. Twenty-three participants met this criterion. Among highly successful learners, 18 (78%) selected a bottleneck location as their first bus-stop choice, again well above chance, $\chi^2(1, N = 23) = 48.79$, $p < 0.001$. Within the group of highly successful learners, eighteen participants rendered the underlying graph perfectly at the end of the experiment, and of these, 17 (94%) chose a bottleneck bus-stop first $\chi^2(1, N = 17) = 58.37$, $p < 0.001$.

Experiment 2

Among single-location trials, we used a Monte Carlo procedure to test for a tendency to select the bottleneck location. Vertex-to-location mappings were repeatedly ($N = 100,000$) randomly permuted within each trial across the entire sample, and for each permutation the number of bottleneck choices was recorded. This resulted in a null distribution of choice frequencies. The result reported in the main text reflects a fixed-effects analysis. In order to test for consistency across the subject population, the same Monte Carlo procedure was used to construct a null distribution for each participant, and a right-sided p-value was derived from this distribution, using the actual number of trials on which the participant selected the bottleneck location. The resulting set of p-values was compared against 0.5 using a Wilcoxon signed rank test. This procedure confirmed a strong tendency, across participants, to select the bottleneck location ($p < 0.003$).

The same analysis strategy was applied for any-order trials, in order to test for a tendency to select the bottleneck location first. Once again, the result reported in the main text reflects a fixed-effects analysis. However, a random-effects analysis based one the same Monte Carlo approach as described for single-location trials indicated a consistent effect across participants ($p < 0.02$).

Experiment 3

A GLM analysis was conducted on log RT data from correct responses, testing for main effects of probe type (bottleneck vs. non-bottleneck) and response (affirm vs. reject). Four further factors were included, in order to assure that any main effect of probe type did not reflect a confound between probe type and

stimulus familiarity. These factors each coded for the cumulative number of occurrences, up to the current trial, of a particular stimulus type. Using the term *multiplicity* for this number of occurrences, the factors were, (1) multiplicity of the current probe, (2) multiplicity of the current start location, (3) multiplicity of the current goal location, (4) multiplicity of the current combination of start, goal and probe. A factor was also included for trial number, and subject was included as a random effect. As reported in the main text, this GLM analysis revealed a main effect of probe type ($F(1, 1474) = 6.838$, $p < 0.01$). A main effect of trial was also observed ($F(1, 1474) = 21.723$, $p << 0.001$). No other main effect reached statistical significance ($p > 0.05$). To check whether the main effect of probe type might reflect a speed-accuracy tradeoff, we compared response accuracy for bottleneck probes against non-bottleneck probes. Mean accuracy was virtually identical for the two conditions (0.967 versus 0.970), a t-test indicated no significant difference ($p = 0.58$).

Experiment 4

Subjects completed an average of 79.24 trials (range 35-148). The trials of interest were those where two shortest-path solutions existed, one of which traversed a single region boundary and the other of which traversed two boundaries (see Figure 2F in the main text). A total of 22 different problems fitting this description occurred during the experiment, all involving shortest-path solutions of either six or seven steps. Twenty-five participants received at least one relevant task assignment. Within this group, the range of such trials per subject ranged from one to seven (median 1.96), with a total sample of 47 trials.

Each trial was classified based on whether the path chosen traversed one or two region boundaries. Thirty-four trials (72%) involved a two-boundary solution. A fixed-effects, one-tailed sign test rejected the null hypothesis that one- and two-boundary solutions were equally frequent ($p = 0.0015$). In order to test for a consistent effect across participants, each participant was classified according to whether he or she more frequently selected a two-boundary solution on trials of interest. Seventeen participants fell into this category, while seven showed the opposite asymmetry (one participant chose one- and two-boundary solutions equally frequently). A one-tailed sign test was consistent with a bias toward single-boundary solutions ($p = 0.032$).
